# Detection of a Frameshift Deletion in the *SPTBN4* Gene Leads to Prevention of Severe Myopathy and Postnatal Mortality in Pigs

**DOI:** 10.3389/fgene.2019.01226

**Published:** 2019-11-26

**Authors:** Martijn F. L. Derks, Barbara Harlizius, Marcos S. Lopes, Sylvia W. M. Greijdanus-van der Putten, Bert Dibbits, Kimberley Laport, Hendrik-Jan Megens, Martien A. M. Groenen

**Affiliations:** ^1^Animal Breeding and Genomics, Wageningen University & Research, Wageningen, Netherlands; ^2^Topigs Norsvin Research Center, Beuningen, Netherlands; ^3^Topigs Norsvin, Curitiba, Brazil; ^4^GD Animal Health Department, Deventer, Netherlands

**Keywords:** animal breeding, loss-of-function, myopathy, pigs, animal welfare

## Abstract

Piglet mortality is a complex phenotype that depends on the environment, selection on piglet health, but also on the interaction between the piglet and sow. However, also monogenic recessive defects contribute to piglet mortality. Selective breeding has decreased overall piglet mortality by improving both mothering abilities and piglet viability. However, variants underlying recessive monogenic defects are usually not well captured within the breeding values, potentially drifting to higher frequency as a result of intense selection or genetic drift. This study describes the identification by whole-genome sequencing of a recessive 16-bp deletion in the *SPTBN4* gene causing postnatal mortality in a pig breeding line. The deletion induces a frameshift and a premature stop codon, producing an impaired and truncated spectrin beta non-erythrocytic 4 protein (SPTBN4). Applying medium density single nucleotide polymorphism (SNP) data available for all breeding animals, a pregnant carrier sow sired by a carrier boar was identified. Of the resulting piglets, two confirmed homozygous piglets suffered from severe myopathy, hind-limb paralysis, and tremors. Histopathological examination showed dispersed degeneration and decrease of cross-striations in the dorsal and hind-limb muscle fibers of the affected piglets. Hence, the affected piglets are unable to walk or drink, usually resulting in death within a few hours after birth. This study demonstrates how growing genomic resources in pig breeding can be applied to identify rare syndromes in breeding populations, that are usually poorly documented and often are not even known to have a genetic basis. The study allows to prevent carrier-by-carrier matings, thereby gradually decreasing the frequency of the detrimental allele and avoiding the birth of affected piglets, improving animal welfare. Finally, these “natural knockouts” increase our understanding of gene function within the mammalian clade, and provide a potential model for human disease.

## Introduction

Piglet mortality is one of the major selection traits in pig breeding and is influenced by the sow, the piglets, and the environment. Hence, piglet mortality is a complex phenotype and depends on the capacity of the sow to raise its offspring, but is also a function of birth weight, management, and selection (Knol et al., 2002). However, also monogenic recessive defects contribute to piglet mortality, although only few examples have been reported in the past (Murgiano et al., 2012; Matika et al., 2019). Even in those cases where the effect of the mutation is severe, selecting efficiently against such a mutation is hampered by the low frequency. In many severe defects, zygotes die very early in gestation, leaving no trace other than the absence of homozygotes in the population at large (Derks et al., 2019).

Inbreeding effects in commercial pig populations are usually kept in check by selective breeding for decreased mortality in piglets by improving both mothering abilities and piglet viability (Olijslagers, 2018). However, variants underlying recessive monogenic defects are not well captured within the breeding values, and potentially drift to higher frequencies as a result of intense selection (Georges et al., 2019). Moreover, those variants can also be maintained as a result of balancing selection for a correlated positive effect in heterozygous state (Derks et al., 2018).

Recessive defects only marginally contribute the overall piglet mortality (Alonso-Spilsbury et al., 2007). Nevertheless, variants affecting piglet mortality are of great importance because those variants directly influence production and animal welfare (Baxter et al., 2013; Rutherford et al., 2013). However, in animal population management, the low-frequency occurrence of defects is usually poorly documented (often very general terms are used), and syndromes are often only recognized once they have reached a high frequency. This is especially relevant for syndromes that do not lead to very distinct phenotypes. Therefore, even in commercial breeding populations little tracking can be done on specific syndromes, and to effectively select against specific low-frequency syndromes therefore requires new approaches.

In this work, we describe the discovery of a highly debilitating syndrome in a commercial pig population through a survey based on a combined medium-density SNP arrays and whole-genome sequencing (WGS). The survey led to the identification of a 16-bp frameshift deletion in the *SPTBN4* gene, with predicted clear phenotypic consequences in homozygotes. The carrier frequency is about 9% in the population under study, affecting approximately 0.81% of the population litters. The frequency was sufficiently low to be unknown to have a genetic basis, and even effectively being unrecognized as a specific syndrome at all. Upon implementation of the survey, one pregnant sow was identified sired by a carrier boar. The affected piglets suffer from myopathy and are unable to walk, usually resulting in death within a few hours after birth, completely in line with predicted pathology in comparison to similar human and mouse cases.

## Material and Methods

### Animals, Genotypes, and Pre-Processing

The dataset consists of 31,839 animals from a synthetic boar line with large white background. The line is maintained and bred in Topigs Norsvin nucleus farms, primarily selecting on production and health traits. The animals were genotyped on the Illumina GeneSeek custom 50K SNP chip (Lincoln, NE, USA). Animals with a frequency of missing genotypes > 0.15 were removed. We discarded markers that did not meet following filtering criteria: A minimum call rate of 0.85, a minor allele frequency > 0.01, and a Hardy-Weinberg proportions exact test p-value below P < 10^−12^. Moreover, markers with unknown location on the Sscrofa11.1 genome build were discarded, leaving 41,573 markers after filtering. All steps were performed in Plink v1.90b3.30 (Purcell et al., 2007).

### Haplotype Phasing and Identification of SSC6 Haplotype

We performed haplotype phasing and imputation of missing sites in Beagle5.0 with parameter for effective population size set to 100, other settings were default (Browning et al., 2018). Expected homozygotes was estimated based on haplotype frequency, using the Hardy-Weinberg principle. An exact binomial test was applied to test the number of observed homozygotes with the number of expected homozygotes. The haplotype was considered significantly depleted if P < 5 × 10^−3^.

### Phenotypic Effects Associated With SSC6 Haplotype

We examined the SSC6 haplotype for records on total number born, number stillborn, mummified piglets, farrowing survival, and lactation survival (survival up to about 21 days of age) of a total of 9,666 litters. We listed these phenotypes for all CxC, and CxN litters identified. We used a Welch’s t-test to assess whether the phenotypes from the CxC litters differ significantly from CxN litters. A p-value < 0.05 was considered significant.

### Whole-Genome Sequencing Analysis and Candidate Variant Identification

The dataset consists of 71 whole genome sequenced individuals from the population under study. All 71 samples were also present in our dataset of 31,839 animals genotyped on the 50K. The 71 samples have a total volume of 1.93 Tbp (tera base pairs) from 14.16 billion 150-bp paired-end reads (**Table S3**). The samples were sequenced on Illumina HiSeq 2000. We aligned the sequences to the Sscrofa11.1 genome build using BWA-MEM version 0.7.15 (Li and Durbin, 2009) with an average mappability of 98.9% and a sample coverage ranging from 8.8 to 14.8X (10.9X average). Samblaster was used to remove PCR duplicates (Faust and Hall, 2014). Samtools was used to sort, merge, and index bam files (Li et al., 2009). Mapping and quality statistics were generated using Qualimap (Okonechnikov et al., 2016). Variant calling was performed with Freebayes v1.1.0 with following settings: –min-base-quality 10 –min-alternate-fraction 0.2 –haplotype-length 0 –min-alternate-count 2 (Garrison and Marth, 2012). Variants with Phred quality score < 20 were discarded (Li et al., 2009). Variants were annotated using the Ensembl variant effect predictor (VEP, release 96) (Mclaren et al., 2016). The impact of missense variants was predicted using sorting intolerant from tolerant (SIFT) (Kumar et al., 2009). LD analysis was performed using Plink v1.90b3.30 (Purcell et al., 2007) with following settings –chr-set 18, –r2, ld-window-r2 0.8.

### SPTBN4 Protein Alignment

Protein alignment between the wild type and mutant protein was performed using ClustalO (Madeira et al., 2019) and visualized using ESPript 3 (Robert and Gouet, 2014). Further visualization and validation was performed using the JBrowse genome viewer version 1.12.1 (Skinner et al., 2009).

### Validation of Causal 16 bp *SPTBN4* Deletion

PCR was done using 60 ng of genomic DNA, with 0.4 µm of each primer, 1.8 mM MgCl2, and 25 units/ml OneTaq^®^ DNA Polymerase (OneTaq^®^ 2X Master Mix with Standard Buffer, New England Biolabs) in manufacturer’s PCR buffer in a final volume of 12 µl. Initial denaturation for 1 min at 95°C was followed by 35 cycles of 95°C for 30 s, 55°C for 45 s, 72°C 90 s, followed by a 5 min extension 72°C. PCR primers for *SPTBN4* are TCAAGGGTGCAGGCTCTTTC forward and GGTAGGAAGCTCGAAGTGGG reverse. The forward primer was dye-labeled with either 6-FAM to produce a fluorescently labeled PCR product detectable on ABI 3730 DNA sequencer (Applied Biosystems). Fragment sizes were determined using GeneMapper software 5 from ABI.

### Histopathological Examination

Two affected piglets less than 1 week old were send to the pathology department of Royal Animal Health (Deventer) for examination. Macroscopically, all observations were within normal limits. Skeletal muscle of the foreleg, the dorsal muscle, and the backside leg of both animals was sampled for routine H&E staining and PTAH staining. The muscle tissue was stored in separate jars and fixated in formaldehyde solution 4%, buffered (=formalin solution 10%, buffered). After that, the tissue was embedded in paraffin and sliced into 2 µm according to standard operation procedure (SOP RAH). Thereafter, the slides were deparaffinized and routinely stained for hematoxylin and eosin (H&E) in an automatic color machine. Simultaneously additional slides of 2 µm of the muscle tissue as well as a positive control slide of muscle tissue were prepared for the manual staining with “phosphotungstic acid hematoxylin,” abbreviated as PTAH. This staining is preferred for demonstrating cross-striations of skeletal muscle.

### Breeding Values and Association Analysis

In this study, we evaluated 63 traits used in the breeding program. Deregressed estimated breeding values (DEBV) were used as a response variable for each trait under study. The estimated breeding value (EBV) of all evaluated traits were deregressed using the methodology described by Garrick et al. (2009). The EBV of each animal was obtained from the routine genetic evaluation by a commercial breeding program (Topigs Norsvin) using an animal model. The reliabilities per animal for the purpose of deregression were extracted from the genetic evaluation based on the methodology of Tier and Meyer (2004). The heritabilities used for the deregression were also extracted from the routine genetic evaluation. Finally, weighting factors based on the estimated reliability of the DEBV were also estimated according to Garrick et al. (2009) using a value of 0.5 for the scalar c. To ensure the quality of the DEBV, only animals with a weighting factors greater than zero and a reliability of the DEBV greater than 0.20 were used in the association analyses. The reliability of the DEBV was also obtained according to Garrick et al. (2009).

Association analyses were performed using the software ASREML (Gilmour et al., 2009) applying the following linear mixed animal model:

$$DEBV_{ij}\omega \;=\;\mu +R_{i}+a_{j}+e_{ij,}$$

where DEBV*_ij_* is the observed DEBV for the animal *j*, w is weighting factor for the residual, *µ* is the overall DEBV mean of the population, *R* *_i_* is the carrier status (count of the detrimental allele) of the *4* mutation *i*, *a* *_j_* is the additive genetic effect estimated using a pedigree-based average relationship matrix, and ** the residual error. Associations with a −log10(P value) greater than five were declared as significant.

## Results

### A 1.5 Mb Segment on Chromosome 6 Affects Lactation Survival in Pigs

We analyzed 31,638 animals from a single purebred boar line (synthetic line with large white background), genotyped on the Porcine 50K SNP chip (Sscrofa11.1 build) (Warr et al., 2019). The analysis revealed a 1.5 Mb segment on chromosome 6 (SSC6:48.75–50.25) showing a deficit in homozygosity associated with reduced lactation survival (**Tables 1** and **2**). The haplotype is segregating at a moderate allele frequency of 4.5% (9.0% carrier frequency) in the population under study. The haplotype frequency has been fluctuating over the last decade, but decreased over the last 3 years (**Figure S1**). We tested whether the frequency was driven by an heterozygous advantage effect. However, we found mostly negative associations with important selection traits except for loin depth and gestation length (**Table 3**), which suggests the frequency is purely the result of genetic drift.

**Table 1:** SSC6 haplotype characteristics.

**Position, Mb**	SSC6: 48.75–50.25
**Number of markers**	19
**Homozygotes expected (HWE)**	61.07
**Homozygotes observed**	0
**Exact binomial test**	3.04e−27
**Carrier frequency %**	9.0
**C x C matings**	52
**Genotyped C x C progeny**	73
**Heterozygote C x C progeny**	46 (63.0%)

Shown are the expected and observed homozygotes for the SSC6 haplotype.

**Table 2:** Carrier-by-carrier litters show 24% decrease in lactation survival compared to carrier-by non-carrier litters. Significant results are indicated in bold.

Status	# Litters	Avg. total born	Avg. live-born	Farrowing survival %	Lactation survival %
NxN	8,105	10.08	9.23	91.37	89.97
CxN	732	10.44	9.62	92.09	90.80
NxC	777	10.16	9.43	92.78	89.85
CxC	52	9.96	9.13	91.82	68.84*

The sow is carrier in CxN litters, while the boar is carrier for NxC litters.

* P < 0.01

**Table 3:** Traits significantly associated with heterozygous carriers of the *SPTBN4* deletion.

Trait	Non-carriers	*SPTBN4* carriers	Effect	SE	−log_10_(P value)
Lactation (pre-weaning) survival^−^	13,789	1,506	−0.39	0.04	20.84
Intramuscular fat (loin)^−^	5,676	696	−0.08	0.01	17.66
Lifetime daily gain^−^	14,089	1,548	−5.62	0.7	14.77
Daily feed intake^−^	14,081	1,548	−16.18	2.99	7.19
Loin depth at end of test period^+^	14,089	1,548	0.28	0.06	6.45
Litter mortality ^−^	13,442	1,462	0.21	0.05	5.25
Gestation length^+^	14,051	1,544	−0.08	0.02	5.04

Effect shows the direction of the association, SE shows the standard error. The symbols “+” and “−” indicate positive and negative effects.

The 52 carrier-by-carrier (CxC) litters show no significant reduction in total number born or liveborn animals. However, lactation survival is reduced by about 24% in CxC litters compared to carrier-by-noncarrier (CxN) matings, indicating that homozygous piglets die within the lactation period (**Table 2**). Next, we examined the remarks for time and cause of mortality of CxC litters. This revealed that most piglets that died within the first 24 h after birth. The majority of those piglets were mostly described by farmers as “weak piglet at birth.”

### Whole-Genome Sequencing Analysis Reveals a 16-bp Frameshift Deletion in *SPTBN4* as the Likely Causative Variant

**Figure 1:**
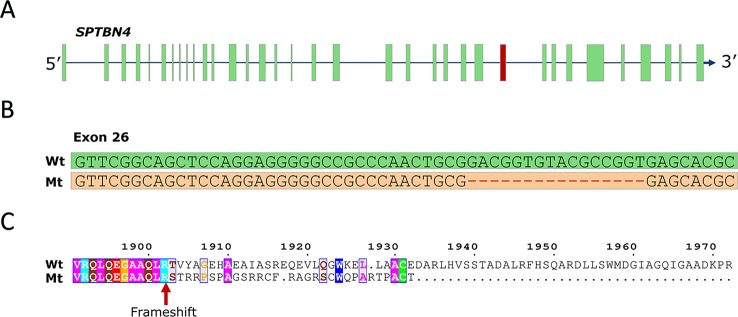
**(A)** SPTBN4 gene model. The location of the affected 26^th^ exon is indicated in red. **(B)** Illustration of the 16-bp deletion. Figure shows wild type and mutant exon. **(C)** Alignment of the mutant (Mt) and wild type (Wt) SPTBN4 protein sequence. The mutation induces 30 novel amino acids and a premature stop codon.

To identify the causal mutation, we examined whole-genome sequence data from 71 animals from the population under study and identified five carrier animals. Linkage disequilibrium (LD) analysis revealed 267 SNP and indel variants in high LD (r^2^ > 0.8) with the SSC6 haplotype (**Table S1**), the majority being in perfect LD (247 variants). Only five variants potentially affect the coding sequence (three missense, one frameshift, one splice-acceptor). The three missense variants are predicted to be tolerated by SIFT (score > 0.18, **Table S1**), while the splice-acceptor variant affects a gene encoding a 28 bp peptide of unknown function, unlikely to be causal. However, one variant in complete LD (r^2 =^ 1) with the haplotype was predicted to have high impact; a 16-bp frameshift deletion in exon 26 of the *SPTBN4* gene (6:g.48801280delGACGGTGTACGCCGGT) (**Figures 1A, B**). The frameshift deletion (ENSSSCP00000031537:p.Arg1902fs) introduces 30 novel amino acids and a premature stop codon, producing an impaired and truncated spectrin beta non-erythrocytic 4 protein (SPTBN4). Mutants lack the final 662 amino acids of the wild type protein (**Figure 1C**), including the pleckstrin homology (PH) domain required for protein transport to membranes (Wang et al., 2018). The SPTBN4 protein is a member of the beta-spectrin proteins and is an actin that links the cell membrane to the actin cytoskeleton. *SPTBN4* mutations disrupt the cytoskeletal machinery controlling proper localization of ion channels in myelinated nerves causing motor neuropathies (Parkinson et al., 2001; Wang et al., 2018).

### Genotyping Five CxC Litters Confirms *SPTBN4* Deletion as the Likely Culprit

We genotyped five CxC litters for the 16-bp deletion which had at least two piglets (range 2–6) that died within the first 48 h after birth. The five litters produced 53 piglets of which 19 were homozygous for the 16 bp deletion (**Table 4**). All 19 homozygous piglets died within 48 h after birth (18 within 24 h). From the 34 remaining piglets (8 wild type, and 26 carriers), only 1 died within 48 h, likely caused by other (environmental) factors.

**Table 4:** Genotyping of the likely causal 16-bp *SPTBN4* frameshift deletion in five carrier-by-carrier litters. The sum per genotype class is indicated in bold.

Litter ID	# Genotyped progeny	# Wild type	# Deletion carrier	# Homozygotes
1	12	3	5	4
2	12	0	8	4
3	11	0	5	6
4	8	1	5	2
5	10	4	3*	3
**SUM**	**53**	**8 (15.1%)**	**26 (49.1%)**	**19 (35.8%)**

All animals homozygous for the deletion died within 48 h after birth.

*One piglet died within 24 h after birth.

### Piglets Homozygous for the *SPTBN4* Deletion Suffer From Myopathy and Hind Limb Paralysis

**Figure 2:**
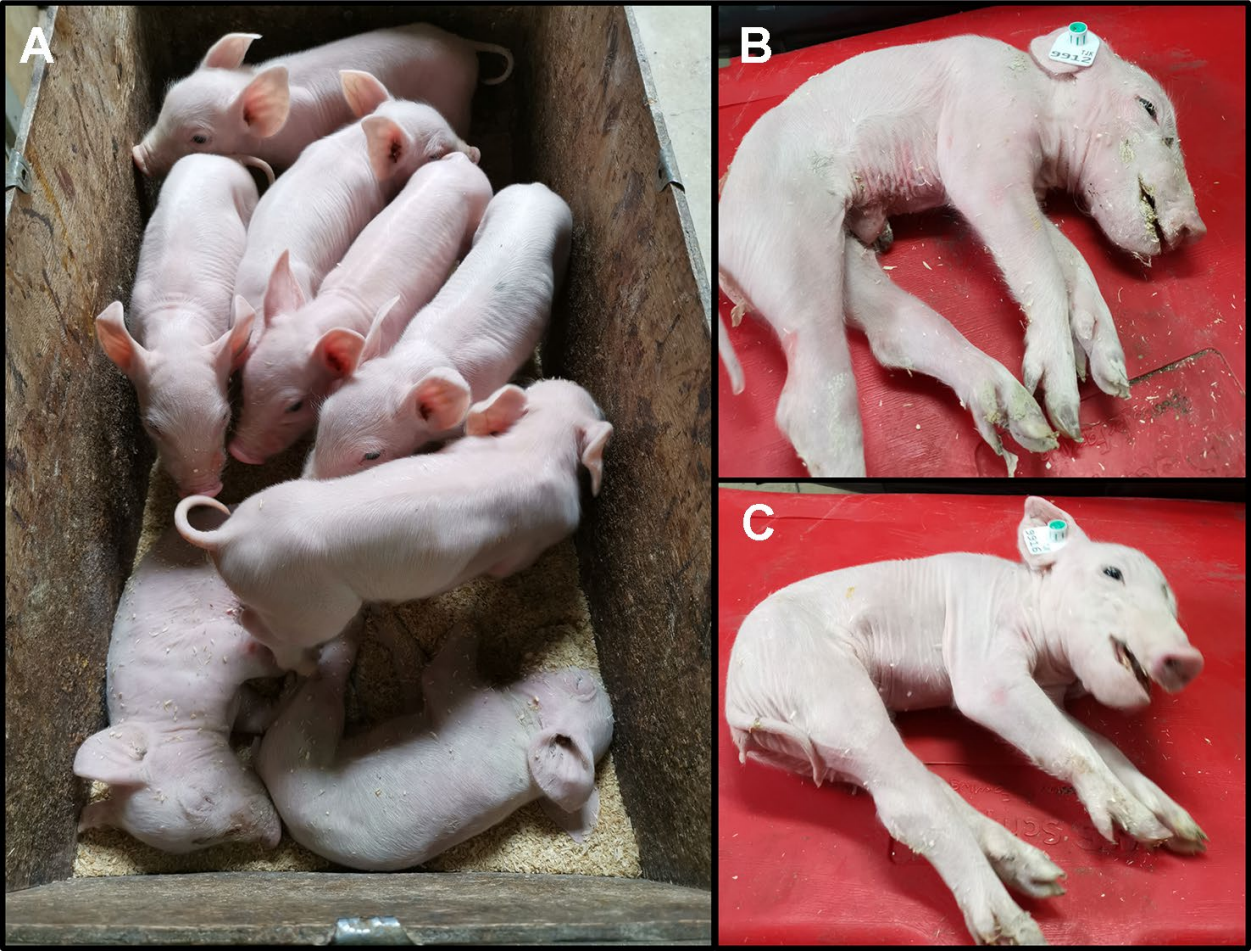
**(A)** Two affected piglets (alive) together with six healthy littermates. The piglets derive from one CxC mating farrowed on 28^th^ of April 2019. **(B)** Affected male piglet 9912. **(C)** Affected female piglet 9916.

We monitored one recent CxC litter (farrowing date: April 28^th^ 2019) that produced six healthy, two affected (samples: 9912, 9916) (**Figure 2A**), and three stillborn piglets. We confirmed the homozygous *SPTBN4* deletion status for the two affected piglets (**Table S2**). Moreover, we observed four heterozygous carriers and two homozygous wild type piglets among the healthy individuals. One of the stillborn piglets (sample: 9921) was also homozygous for the deletion, while the other two were heterozygous. The affected piglets suffer from extreme muscle weakness (**Figures 2B, C**), paralysis of the hind limbs, and tremors (**S1 Video**). Hence, the piglets were unable to walk or drink.

#### Affected Piglets Lack Cross Striations in the Dorsal and Hind Limb Skeletal Muscles

**Figure 3:**
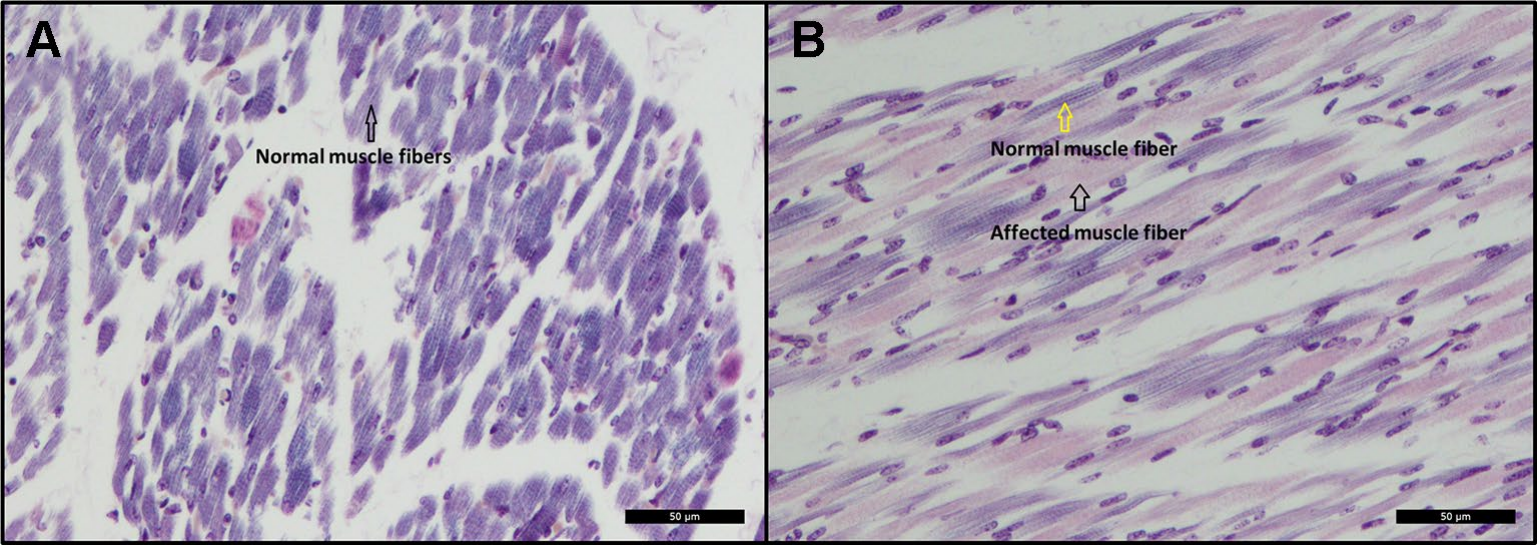
**(A)** Cross-sectional view of a skeletal muscle from the front leg. The black arrow indicates normal coloring (dark) of muscle fibers indicating presence of cross-striations. PTAH Bar  =  50 µm. **(B)** Cross-sectional view of a skeletal muscle from the hind leg. The black arrow indicates abnormal coloring (pink) of muscle fibers indicating lack of cross-striations. The yellow arrow indicates normal coloring and presence of cross-striations. PTAH Bar  =  50 µm.

Histopathological examination revealed scattered degeneration of muscle fibers in both piglets, and focally necrosis and vasculitis in the dorsal muscle in one of the piglets (ID = 9912). Moreover, phosphotungstic acid hematoxylin (PTAH) staining shows divergent coloring of the skeletal muscle fibers, indicating decrease of cross-striations, particularly in the muscles of the dorsal and hind legs of the affected animals (**Figure 3B**), while the front legs seem unaffected (**Figure 3A**). The decrease of cross striations is indicated by abnormal coloring and general loss in volume of muscle fibers (**Figure 3B**). The histopathologically observed changes in the hind legs and in the dorsal muscles are indicative for muscular dystrophy.

## Discussion

In this work we report a novel congenital defect causing piglet mortality likely due to a 16 bp frameshift deletion in the *SPTBN4* gene. The piglets suffer from extreme muscle weakness (myopathy) and die within a few hours after birth. The deletion is expected to confer a complete loss-of-function of the spectrin beta, non-erythrocytic 4 protein. *SPTBN4* is a member of the family of spectrin genes and is required for ion channel clustering at the nodes of Ranvier, affecting action potential (Devaux, 2010). Mutations disrupt the cytoskeletal machinery that controls proper localization of ion channels and function of axonal domains mainly at the axon initial segments (AIS) and the nodes of Ranvier (Wang et al., 2018). More specifically, the affected C-terminal domain of SPTBN4 is crucial for KCNQ2 channel trafficking and excitability at nodes of Ranvier (Devaux, 2010).

Subsequent follow-up research identified human and mouse cases that indicated that the ensuing syndrome would likely not prove to be immediately lethal, but rather confer severe myopathy. By medium-density SNP genotype data, available for all animals in the breeding population (N = 31,839), carriers could be identified. Among those carriers was a sow that was approximately mid-term in pregnancy at the time of identification, sired by a boar that was also carrier. The breeding farm was notified to document the litter at birth. The observed phenotype of the affected piglets (myopathy, hind limb paralysis, tremors) was completely congruent with what was observed in human patients with homozygous loss-of-function or compound heterozygous mutations in the *SPTBN4* gene (OMIM: 606214). Two of the human patients have loss-of-function mutations within the PH domain (Wang et al., 2018), supporting that a loss of the PH domain in pigs would likely lead to a complete loss-of-function of the SPTBN4 protein. In human, similar mutations lead to severe congenital myopathy caused by the absence of muscle type I fibers, neuropathy, and deafness (Knierim et al., 2017; Wang et al., 2018). Wang et al. (2018) also observed motor axonal neuropathy in several patients characterized by congenital hypotonia, profound weakness, and loss of deep tendon reflexes by early childhood. Moreover, nerve biopsies revealed reduced nodal Na+ channels and no nodal KCNQ2 K+ channels, revealing the molecular pathology causing nervous-system dysfunction. Therefore, we conclude that this frameshift variant is the likely causal mutation leading to the observed phenotype and depletion of the homozygous genotype in the population. Future studies could focus on making an *in vivo* knockout of the *SPTBN4* gene in pig, to study the syndrome and associated phenotype in more detail.

We did not observe degeneration of muscle fibers in the front legs, while the dorsal and hind leg muscle fibers were clearly affected. This observation could partly explain the hind limb paralysis, while the front legs are not affected. The discrepancy between front and hind legs muscle fibers has also been described in quivering mice, in which *SPTBN4* loss-off-function mutations cause motor neuropathy, hind limb paralysis, tremors, and central deafness (Parkinson et al., 2001; Komada and Soriano, 2002). Parkinson et al. (2001) describe reduced nerve-conduction velocities in sciatic nerves of mice with quivering alleles causing the peripheral hind limb neuropathy. Expression of *SPTBN4* in mice is restricted to the brain, spinal cord, and sciatic nerves and not observed in skeletal muscle, so this disease is primarily a neuronal defect. Overall it remains unclear which mechanism causes the absence of symptoms in the forelimbs. This “natural knockout” in pigs can be a useful resource to study the human disease, as pigs are usually a better model to study human disease compared to rodent species. Moreover, the consequence of the loss of *SPTBN4* function can be studied in more detail.

The effective population size (Ne) of the breed under study is estimated to be around 100 (Hidalgo et al., 2016). In animal breeding, low Ne increases the risk that detrimental alleles rise in frequency by chance. Moreover, previous studies have shown that recessive lethal alleles can be driven by advantageous effects in heterozygotes (Derks et al., 2018; Matika et al., 2019). Matika et al., 2019 found a recessive stop-gained mutation in the *MSTN* gene associated with a major increase in muscle depth in heterozygotes. However, we find no evidence for any heterozygous advantage in our study. With the current genomic techniques we can now identify deleterious alleles drifting to higher frequencies, and monitor the emergence of novel deleterious alleles accurately, allowing more effective purging. Moreover, the result of this type of study will greatly improve the consciousness of “hidden” genetic defects at both the breeder and farmer level. Without any prior information, rare birth defects are often recorded as “weak piglet.” And without any further distinction of specific syndromes, further action is not possible. In most cases it is unknown if there is a genetic basis, or that there may be other confounding effects. With prior genomic information, the syndrome can be identified, compared to other cases, and carriers identified, leading to actionable information.

Piglet mortality is of high economic and animal welfare importance. Hence, the discovery of the *SPTBN4* mutation has led to immediate implementation in the breeding program to minimize the frequency of carrier-by-carrier matings. This enables to avoid the birth of affected individuals, thereby improving animal welfare and reducing economic losses.

## Conclusion

In this study we report a novel congenital defect likely caused by a recessive frameshift deletion in the *SPTBN4* gene in pigs. The findings are supported by striking similarities to *SPTBN4* associated syndromic phenotypes in humans and mice. The study allows to monitor and purge the deleterious allele from the population. Carrier-by-carrier crosses can be prevented, precluding affected individuals, thereby reducing economic losses, and improving animal welfare. Finally, these “natural knockouts” obtained in the breeding industry can provide a model for human disease and increase our understanding of gene function within the mammalian clade, and provide a potential model for human disease.

## Data Availability Statement

50K Genotypes, WGS variants (VCF) and alignment files (BAM) are available at the Open Science Framework repository: https://osf.io/em2db/?viewonly=f324f84b45014e14a9a4839c32ec7a27under DOI:10.17605/OSF.IO/EM2DB.

## Ethics Statement

Ethical review and approval was not required for the animal study because the data used in this study has been obtained as part of routine data collection from Topigs Norsvin breeding programs, and not specifically for the purpose of this project. Therefore, approval of an ethics committee was not mandatory. Sample collection and data recording were conducted strictly according to the Dutch law on animal protection and welfare (Gezondheids- en welzijnswet voor dieren). Written informed consent was obtained from the owners for the participation of their animals in this study.

## Author Contributions

MG, H-JM, and MD conceived and designed the study. BH was responsible for general organisation and communication with Topigs Norsvin and farmers. MD and ML performed the data analysis. BD and KL performed lab work. SG-V performed the pathological analysis. MD wrote the manuscript. H-JM, MG, BH, SG-V, BD, KL, and ML provided useful comments and suggestions and helped to draft the manuscript. Phenotypic data was analysed by ML. All authors read and approved the final manuscript.

## Funding

This research was funded by the STW-Breed4Food Partnership, project number 14283: From sequence to phenotype: detecting deleterious variation by prediction of functionality. This study was financially supported by NWO-TTW and the Breed4Food partners Cobb Europe, CRV, Hendrix Genetics and Topigs Norsvin. In addition, this study was supported by the IMAGE project (Horizon 2020, No. 677353). The funders had no role in study design, data collection and analysis, decision to publish, or preparation of the manuscript. The use of the HPC cluster was made possible by CATAgroFood (Shared Research Facilities Wageningen UR).

## Disclaimer

The data used in this study has been obtained as part of routine data collection from Topigs Norsvin breeding programs, and not specifically for the purpose of this project. Therefore, approval of an ethics committee was not mandatory. Sample collection and data recording were conducted strictly according to the Dutch law on animal protection and welfare (Gezondheids- en welzijnswet voor dieren).

## Conflict of Interest

ML and BH are employees of Topigs Norsvin Research Center, a research institute closely related to one of the funders (Topigs Norsvin).

All the remaining authors declare that the research was conducted in the absence of any commercial or financial relationships that could be construed as a potential conflict of interest.

The other Breed4Food partners Cobb Europe, CRV, Hendrix Genetics, declare to have no competing interests for this study.

## Supplementary Material

Video S1 Video showing both affected individuals after birth.

Table S1 Genomic variation in high LD with the SSC6 haplotype.

Table S2 Genotyping of the CxC litter including two affected individuals (farrowing date: April 28th 2019). Table shows homozygous del/del status for the two affected piglets 9912,9916.

Table S3 Mapping and coverage statistics from WGS samples.

Figure S1 SPTBN4 deletion carrier frequency from 2007–2019.
