# Supplementary figures and images for: Detection of a Frameshift Deletion in the SPTBN4 Gene Leads to Prevention of Severe Myopathy and Postnatal Mortality in Pigs
Source: Front Genet. 2019 Nov 26;10:1226. doi: 10.3389/fgene.2019.01226 (PMC6902008; doi:10.3389/fgene.2019.01226)

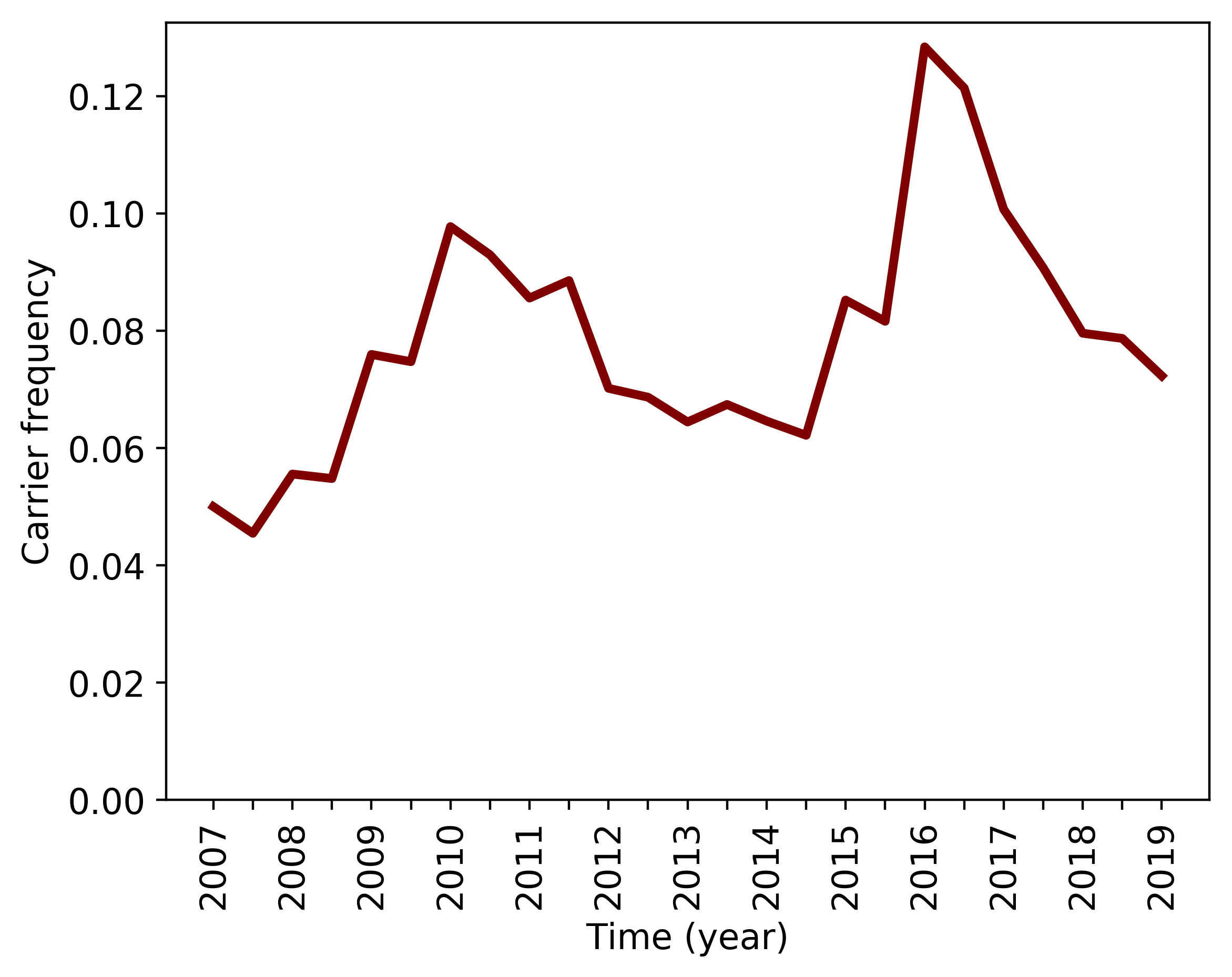

Supplement: Figure S1 — SPTBN4 deletion carrier frequency from 2007–2019. [file Image_1.png]
